# Analysing lapsing rates among first-time blood donors at a blood centre in Zimbabwe using survival analysis

**DOI:** 10.11604/pamj.2024.47.211.39015

**Published:** 2024-04-25

**Authors:** Delson Chikobvu, Coster Chideme

**Affiliations:** 1 Department of Mathematical Statistics and Actuarial Sciences, University of the Free State, Bloemfontein, South Africa

**Keywords:** Survival analysis, Kaplan-Meier method, Cox proportional hazard model, hazard ratio, log-rank test, survival function

## Abstract

**Introduction:**

blood centres are often faced with the problem of donor lapsing resulting in loss of donors from the already strained donor pool. In Zimbabwe, 70% of the donated blood comes from younger donors aged 40 years and below, who at the same time, have high attrition rates. This study seeks to apply the concept of survival analysis in analysing blood donor lapsing rates.

**Methods:**

in analysing the donor lapsing and retention rates, data on 450 first-time blood donors at the National Blood Service Zimbabwe, in Harare´s blood bank for the period 2014 to 2017 was extracted from the donors´ database. The Cox proportional hazards (Cox PH) and Kaplan-Meier methods were applied in the analysis. Donor demographic characteristics suspected of having effect on donor lapsing and retention were identified and analysed.

**Results:**

the study findings show that 56.9% of the donors had lapsed by the end of the four-year study period. Results from the multiple Cox PH model indicate that donor age had a significant effect on blood donor retention time (p = 0.000918 < 0.05). The hazard ratio (HR) = 0.615 with 95% CI: (0.461; 0.820) shows that the relatively older donors had a lower hazard (38.5% lower) of lapsing compared to the hazard for younger donors. The effect of gender, blood donor group and donation time interval on donor retention and attrition were not statistically significant. Male donors had HR = 1.03; 95% CI (0.537; 1.99) with (p = 0.922 > 0.05) and donors with a 4-month interval between donations had HR = 1.31; 95% CI (0.667; 2.59) with (p = 0.430 > 0.05).

**Conclusion:**

the study confirmed the problem of donor attrition faced by blood centres. The age of the donor had a significant effect on the retention time of blood donors before lapsing. The older the blood donor, the lower the risk of lapsing. The Zimbabwe National Blood Service (NBSZ) Blood Centre authorities should have a critical mass of individuals above 40 years as potential blood donors because of their reliability in blood donation according to the study findings.

## Introduction

The discrepancy between blood supply and demand in blood transfusion services is becoming a major public health issue globally. The problem is being worsened by a decline in the number of voluntary and regular blood donors. Blood centres are often left with no other choice but to depend upon a small proportion of the eligible population that donates blood to meet all the blood requirements [1]. In Zimbabwe, over 70% of the blood is donated by young donors below the age of 40 years, who on the other end, have high attrition rates. This phenomenon is common in many blood centres where most first-time blood donors lapse during the first year of their donation career [2].

Blood donor lapsing often results in a two-pronged problem, viz; high costs of donor replacement and blood shortages during disasters when there is a sudden surge in demand for blood. The analysis of trends in donor lapsing helps blood managers in decision-making on when to conduct blood donor recruitment drives and the specific target groups of donors. First-time blood donors supply most of the blood in blood centres, hence the need to encourage them to become regular donors and minimise lapsing rates [3,4].

In survival analysis, the survival time is defined as just time in this study, starting from the entry into observation, up to the time of occurrence of some event [5]. In the context of this study, survival times inform/measure the follow-up time from the day a donor makes a first-time blood donation, up to the time the donor lapses (occurrence of the event). The event of occurrence in the study is defined as a blood donor lapse (equivalent to the death event in survival analysis). Blood donor lapse is defined as not making a blood donation for a period of 24 consecutive months from the last donation. The assumption is that once a donor has lapsed, he/she is lost from the donation pool. The lapsed state is considered to be an absorbing state. After the first-time donation as a new donor, donors can transit into a regular, occasional or lapsed state. Donor retention or survival is defined as preventing donors from lapsing and eventually becoming inactive.

Survival analysis is a useful tool in analysing the length of time a blood donor is active during their donation career [6]. Survival analysis techniques have been widely used in the healthcare sector in areas such as human organ transplants, cancer studies and other disease progressions with emphasis on the effects of various factors on patient survival times [7-9]. According to a study at the Gulf Coast Regional Blood Centre in the United States of America (USA), the Kaplan-Meier estimates derived from the survival function of first-time donors, indicated high donor return rates after a year [10]. The log-rank test analysis showed a significant difference in return rates by blood type.

A study on blood donors from Ribeirão Preto Blood Centre in Brazil focused on the return rate of first-time blood donors [11]. The study used a parametric long-term survival model to estimate the proportion of donors who never returned for further donations. From the analysis, using the Kaplan-Meier method, the authors concluded that a significant proportion of first-time donors rarely return for a second donation. The study also concluded that there were no statistically significant differences in lapsing rates among donor characteristics such as gender and blood group. In another study, time intervals between donations were modelled using an extended Cox proportional hazard model [12]. The study established that, the donor's socio-demographic characteristics such as age, weight and occupation had significant effects on the time interval between donations. In a related study, the Cox proportional hazard regression model was used to analyse factors with effect on blood donation intervals [13]. The model indicated a significant association between donor marital status and the time interval between donations, with higher return rates for married donors.

Survival analysis techniques were also applied to analyse blood donor return behaviour [14]. From the proportional hazard models, survival curves and relative risk were estimated. The study evaluated the effect of donor demographic characteristics on the probability of repeat blood donation. In a blood supply management study at Lansing Centre of the Great Lakes Region in the USA, survival analysis methods were applied to analyse differences in blood donation processing time among different blood donor classes [15]. The study concluded that there was no significant difference between first-time and regular donors in donation processing times.

In a study conducted on donor lapsing, several factors which affect donor lapsing were identified, and donor deferrals and adverse events reactions had the most effect on donor attrition rates [16]. The study also established that older male donors had a lower likelihood of lapsing and hence were likely to return for further donations.

The sections in this paper are organised as follows, section 2 presents data collection and the methodology. Section 3 focuses on data analysis and results. Section 4 presents discussions and conclusions are in section 5.

## Methods

**Study design:** a retrospective study was conducted to determine the effects of blood donor-specific characteristics such as gender, age, blood group, and time interval between donations on donor lapsing and retention rates at a selected blood bank in Zimbabwe.

**Study setting and population:** Zimbabwe is located in sub-Saharan Africa, bordering South Africa to the south, Mozambique to the east, Zambia to the north, and Botswana to the southwest. Zimbabwe is divided into 10 administrative provinces including Harare and Bulawayo being the capital and second capital cities respectively. According to a national population census conducted in the year 2022, the population of Zimbabwe was estimated to be around 15 million. The proportions of the population based on gender were found to be 52% females and 48% males.

The study was based on a sample of blood donation data collected at a blood centre in Harare, Zimbabwe. The time period for the study spans across four years (48 months), from 1^st^ January 2014 to 31^st^ December 2017. The NBSZ Harare Blood Centre was chosen as the study site since it is the head office and largest bank of the blood service, where all the blood donation data in Zimbabwe is collated and managed. The donation data is assumed to contain a diversity of characteristics of the blood donors in Zimbabwe´s donor population.

The study population was voluntary non-remunerated new blood donors. A new blood donor is defined as a blood donor giving blood for the first-time in their lifetime. To qualify as a blood donor, one is expected to be at least 16 years old and weigh more than 50 kilograms.

**Variables:** the outcome of interest is the time from the first donation to lapsing. In this study, the time variable was recorded in months and the event variable was a lapsed blood donor, otherwise, the donor is deemed to be an active donor. Active status means that the donor was still donating blood (or expected to donate blood), while lapsed status means that the donor was no longer considered to be donating blood and was out of the donor pool. The data was right censored as a consequence, accounting for the donors who did not lapse during the period of the study.

A number of covariates are deemed to be factors that influence the retention of the donor during the donation career. The study focused on the following factors, viz; age, blood group, gender, and time interval between donations.

**Description of variables:** time: {time from first-time donation to lapsing}; status: {0 = censored (donor not yet lapsed when the study ends), 1 = blood donor lapsed within some time interval before end of the study}; donor age group (D Age): {0 = age < 40 years; 1 = age ≥ 40 years}; gender (sex): {0=male, 1=female}; blood group: {group O=0, other blood groups=1}; time interval between donations group: {0= 3-months, 1= 4-months}.

### Data sources and measurement

**Data collection tool:** an Excel sheet record file was created by the NBSZ data capture clerk on request by the researcher and the data was entered under various headings.

**Data collection:** the donors´ specific data on the date of first donation, date of last donation, age at first donation, gender, number of donations each year, time interval between blood donations and blood group were extracted as secondary data from the blood centre´s database. Time intervals between donations were determined from the blood service´s guidelines. In Zimbabwe, blood donation frequencies vary by gender, with females at pre-menopausal donating after every 4 months (16 weeks or 120 days), females post-menopausal and males can donate after every 3 months (12 weeks or 90 days). Furthermore, the identity of the blood donors remained anonymous, only identification numbers were used for each donor. The authority to use the data was granted by the NBSZ Research and Development Department.

**Sample size:** the sample size was calculated using the Taro Yamane formula [17] which is stated as:


$$n=\frac{N}{\left(1+N*E^{2}\right)}$$

Where found to be 382 and was increased by E is the error in the calculation (95% or 0.05). A total of all the 8312 first-time voluntary and non-remunerated blood donors in 2014 were retrieved from the donors´ database thus forming the study population. From the Taro Yamane formula, the sample size was found to be 382 and was increased by an additional 68 donors to make it 450 donors. This was to improve the accuracy of the estimates. Random sampling was then used to select the objects of the study.

**Statistical methods:** donor retention or survival is defined as preventing donors from lapsing and eventually becoming inactive. Comparisons on the effects of subgroups of donor-specific characteristics on donor retention times are also made. Once a donor was lapsed, no further donations were expected from the donor since the lapsed state was deemed to be an absorbing state. Furthermore, the data had censored observations. At the end of the study, some blood donors had experienced the event (lapsed) and other donors had not yet experienced the event (not lapsed).

The Kaplan Meier, the log-rank test, and the Cox Proportional hazard model were applied in the analysis to understand the influence of covariates on donor lapsing. The log-rank test was used to compare donor retention rates between subgroups for each covariate. The Cox PH model was used to select significant risk factors that had the most impact on donor retention rates. Donor-specific covariates such as gender, age, blood group, and the time interval between donations were analysed in order to establish if they had effects on donor lapsing. The survival analysis packages built in R software version 4.0.2 were employed in the analysis.

**Justification of the use of survival models:** the possibility of following up with a blood donor from the day of first-time blood donation, up to the time the donor lapses (event occurrence) is important in survival model application. It is common that by the end of the study, some blood donors would have experienced the event (donor lapsed) and other donors may not yet have experienced the event (not lapsed thus allowing for data censoring). These scenarios justify the application of survival models in blood donation. Survival analysis is a useful tool in analysing the length of time a blood donor is active during their donation career (the survival equivalent).

**Model formulation:** it is possible to determine the time to event since the date at which the first donation was made is recorded and the donor´s donation history can be followed up from the records until the time the last donation was made. If there is no donation for the past 24 months, the donor is treated as lapsed. Therefore, the exact date of lapsing was inferred from the date of the last donation. It is assumed that the donor lapsing event has occurred at 24 months after the last donation for purposes of this study and is also applied at the National Blood Service, Zimbabwe.

The time T, that a blood donor lapses, can be regarded as a random process that depends on various covariate factors. Let the length of time until the event takes place be T, such that T ≥ 0.

The probability of the event taking place is a function of time expressed as:


$$T=t_{k}-t_{i}\;\left(1\right)$$

Where: t_k_is the time when the event occurred; t_i_is the time first exposed to the risk of the event taking place; f(t) is the density function of the duration T; F(t) is the cumulative density function of the duration T. Now:


$$F\left(t\right)=P\left(T\leq t\right)=\int_{0}^{t}\left(u\right)du\;\left(2\right)$$

$$f\left(t\right)=F'\left(t\right)\;\left(3\right)$$

Equation (2) and equation (3) can be used in the graphical representation of donor lapsing (risk or event) against time (T) during the blood donation career. This graph would show the probability of lapsing varying with time from the point of first blood donation.

**Data censoring:** taking cognisance of the study duration, it is possible that some donors may not lapse during the study time period, thus producing the right-censored observations. Thus, right censoring may arise in the following scenarios: i) A blood donor has not yet experienced the event (lapsed) within the study duration; ii) a blood donor is lost to follow-up during the study period; iii) a donor experiences a different event that makes further follow-up impossible.

The three scenarios above are typical examples of right censoring applicable in survival analysis [18]. The survival analysis technique is premised on two critical functions which describe the probability of an event taking place, viz; survivor and hazard functions.

**Survivor function:** the survivor function, S(t), is the unconditional probability that the event is yet to take place at time t. In the context of this study, the survivor function represents the probability that the blood donor has not yet lapsed at time t. It can also be described as the probability that a blood donor does not lapse from the first-time donation to a specified future time t. Its emphasis is on not experiencing the event of interest. The survivor function can be expressed as follows [19]:


$$\begin{array}{ll} S\left(t\right) & =P\left(T\geq t\right)\\ & =\int_{t}^{\infty }f\left(u\right)du\\ & =1+P\left(T\leq t\right)\\ & =1-F\left(t\right),\text{ using Equation }\left(2\right). \end{array}\;\left(4\right)$$

**Hazard function:** hazard refers to the rate at which a randomly selected blood donor, known to be active at the time (t - 1), will lapse at time t. The hazard function refers to the probability that a process that has been ongoing until time t will experience an event in time period t. In the context of this study, the hazard function represents the probability that a blood donor who has been donating blood for time period t (which can be months or years), would be lapsing now. The hazard function can be expressed as follows [19]:


$$h\left(t\right)=\underset{\Delta t\to 0}{\lim}\frac{P\left(t\leq T<t+\Delta t|T\geq t\right)}{\Delta t}\;\left(5\right)$$

It is the probability of lapsing in a small period between t and (t + Δt) given that the donor has survived up until time t. For a blood donor D_i_


$$S\left(t,D_{i}\right)=P\left(T_{i}\geq t\right)=1-F\left(t,D_{i}\right)\;\left(6\right)$$

Where T_i_is time of lapsing. The risk of donor lapsing at time t is:


$$h\left(t,D_{i}\right)=\underset{\Delta t\to 0}{\lim}\frac{P\left(T_{i}\leq t+\Delta t|T_{i}\geq t\right)}{\Delta t}\;\left(7\right)$$

$$=\frac{f\left(t,D_{i}\right)}{S\left(t,D_{i}\right)}$$

Hazard is a measure of risk. Greater hazard implies a higher risk of lapsing in the time interval T_i_.

**Kaplan-Meier estimation:** the Kaplan-Meier (KM) is a non-parametric estimation method for survivor function which is performed at the beginning of the analysis to understand the trend of survival probabilities [18]. The survival function from the KM estimator is represented by a series of declining horizontal steps. The value of the survival function between successive observations is assumed to be constant. Each step corresponds to the occurrence of an event or events. The KM survival function can be expressed as follows [18]:


$$S\left(t_{i}\right)=S\left(t_{i-1}\right)\left(1-\frac{d_{i}}{n_{i}}\right)\;\left(8\right)$$

Where: S (t_i_) = probability of surviving at time t_i_; S(t_i-1_) is the probability of surviving at time t_i-1_; d_i_ is the number of donors having event of interest (lapsing) at time t_i_; n_i_ is the number of donors surviving before t_i_.

When the study begins at time zero, all the donors are active and the survival probability is S(0) = 1. The estimated survival probability only changes at the time t_i_, of the event of interest (donor lapsing), and it is constant between the two times (t_i_ and t_i+1_). Under the KM technique [20], the subjects that are censored, are assumed to have equal probabilities of surviving, as those remaining in the study. In other words, the KM estimator is a step function with jumps at the observed event times and the jumps depend on both the number of events observed at each event time t_i_ and the pattern of the censored observations preceding t_i_.

**Cox-proportional hazard regression model:** the Cox PH model is suitable for finding the correlation between explanatory variables with the survival or any right-censored response variable [19,21,22].

The Cox PH model is a powerful technique for analysing survival data and identifying covariates that affect survival. Unlike the Kaplan-Meier which only uses one variable for estimating the survival curves, the Cox PH model uses multiple independent variables for estimating the differences between the survival curves. Through the application of the Cox PH model, the effects of the various donor-specific factors on the hazard are determined. Under this model, censoring occurs randomly, the time is continuous and the hazard function for two subjects relative to covariate subjects is independent of time.

The Cox PH model is expressed as follows [19,21]: For a blood donor D_i_


$$h_{i}\left(t,D_{i}\right)=h_{0}\left(t\right)\exp\left(\beta x_{i}^{\tau }\right);i=1,2,3\ldots n\;\left(9\right)$$

h_i_(t, D_i_) is the hazard calculated for each blood donor, and h_0_(t) is the baseline hazard. X_i_= (X_i1_, X_i2_, X_i3_,..., X_in_) is the vector of variable factors. β = (β_1_, β_2_, β_3_, ..., β_n_) is the vector of regression coefficients and τ transposes the vector.

**The proportional hazard (PH) assumption:** the assumption states that the hazard functions are multiplicatively related and their ratio is constant over survival time. This means that the estimated hazard ratios do not depend on time. If this assumption is violated, then the results of the analyses may be misleading and this could lead to wrong conclusions.

**Test for PH assumption:** when the parameter estimates β_1_, β_2_, ..., β_q_ do not vary much over time, proportional hazards can be assumed. The hypothesis tested is then stated as: H_0_: regression coefficients are constant over time.

**Test of goodness of fit:** the log-rank test is popular in testing the significant difference among various survival curves and is an approximation of the Chi-square test. It tests for the existence of significant differences in survival time between different independent categories under study. In other words, it is used to examine the association between the length of retention of a blood donor (how long they are retained before lapsing) and whether or not age, gender, and the interval between donations and blood group have significant effects.

**Comparison of the survival estimates across groups:** the trends exhibited by Kaplan Meier curves are further analysed using the log-rank test and Peto-Peto test for comparing survival times between groups. The Peto-Peto test will be compared against the log-rank test to check the accuracy of the survival estimates. A parameter rho; ρ = 1 was used in the Peto-Peto test. ρ is a scalar parameter that controls the type of test. ρ = 0 gives the log-rank or Mantel-Haenszel test, and ρ = 1 is equivalent to the Peto & Peto modification of the Gehan Wilcoxon test.

**Ethical consideration:** the blood donation data used in this study were as approved by the General/Human Research Ethics Committee (GHREC) of the University of the Free State, South Africa (Ethical Clearance number: UFS-HSD2023/1370).

## Results

**Descriptive analysis:** Table 1 summarises the frequencies of lapsing donors for the period 2014 to 2017 (study period) extracted from the donors´ database.

**Table 1:** descriptive statistics of lapsing blood donors

	Subgroups	Lapsed donors	Percentage (%)
Status	Lapsed	256	56.9
	Active	194	43.1
	Total	450	
Age	< 40 years	186	72.7
	≥ 40 years	70	27.3
Gender	Male	167	65.2
	Female	89	34.8
Blood group	Group O	124	48.4
	Other groups	132	51.6
Interval between donations	3 months	175	68.4
	4 months	81	31.6

Results from Table 1 show that 56.9% of the donors experienced the event (lapsed) leaving 43.1% of the donors were still active by the end of the study. The fact that more than half of the donors lapsed within a period of four years reveals the concerns around donors lapsing in blood establishments. The data analysis shows that the younger blood donors (< 40 years) account for 72.7% of the lapsed donors leaving 27.3% of the lapsed blood donors as the older donors (≥ 40 years). The study sample had more male donors than females, this then translated to a higher proportion (65.2%) as male donor lapses compared to 34.2% for the females. However, this did not necessarily mean males were more likely to lapse than females. Considering the male donors alone, 167 males out of 308 males lapsed representing 54.2% of lapsed males. At the same time, 89 out of 142 females lapsed, and this represents 62.7% of lapsed females.

**Figure 1:**
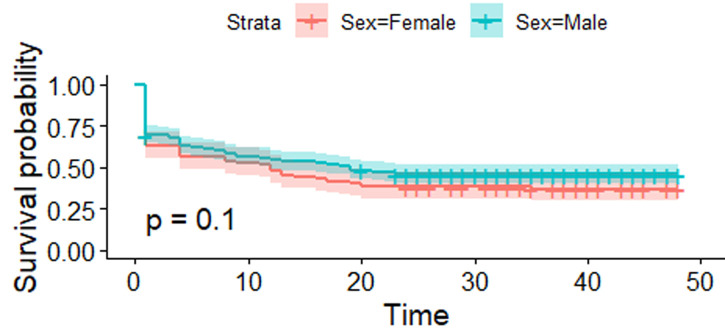
estimated survival (retention) functions for male and female blood donors

**Main results:** Figure 1 shows the estimated survival curves for the male and female blood donors with their confidence intervals. Censored observations were plotted as a plus (+) sign. With a p-value = 0.1 > 0.05, the survival functions are not statistically significant between male and female donors. This means the survival (retention) functions are the same for male and female donors.

**Figure 2:**
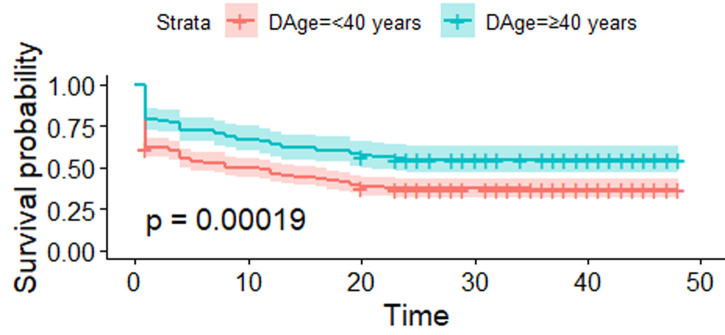
estimated survival (retention) functions for younger and older blood donors

Figure 2 shows the estimated survival curves for younger blood donors (< 40 years) and older blood donors (≥ 40 years) with their confidence intervals. The p-value = 0.00019 < 0.05 shows that the age subgroups differ significantly in survival (retention). The survival function curves show that a significant decline in survival probability is experienced faster within the first twenty months of blood donation for younger donors.

**Figure 3:**
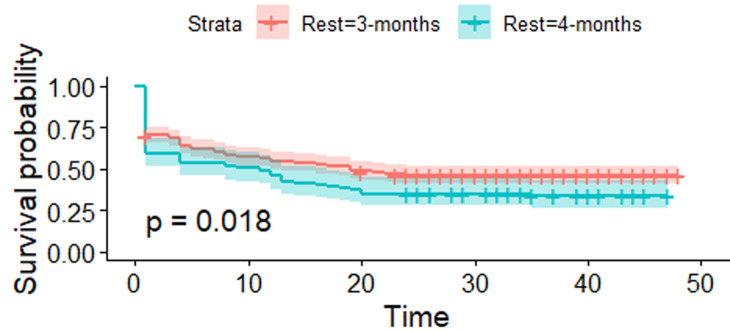
estimated survival (retention) functions for intervals between donations

**Figure 4:**
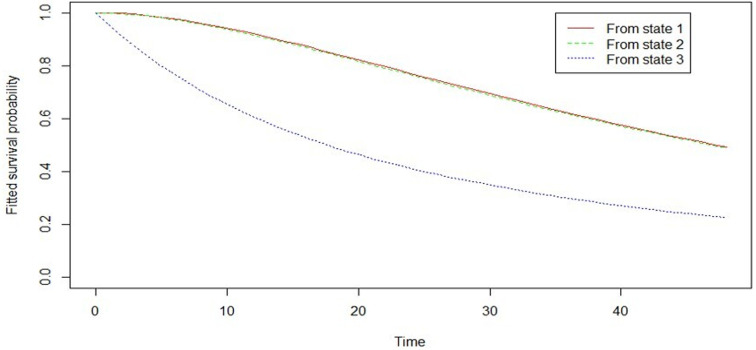
estimated survival (retention) functions from each state of blood donation

Figure 3 shows the estimated survival (retention) curves for the interval between donations and their confidence intervals. The p-value = 0.018 < 0.05 shows that the survival functions are not the same between 3-month and 4-month intervals between blood donations. This means the risk of lapsing for the interval between donation subgroups is statistically different. Figure 3 also shows that, beyond 24 months, the survival probabilities stabilise thus further confirming the trend of high attrition rates during the first year of blood donation. The higher likelihood of lapsing as a result of erratic blood donations is further demonstrated in Figure 4.

Figure 4 illustrates survival functions from each of the three states of blood donation viz; new (state 1), regular (state 2), occasional/erratic (state 3). The plot shows that the probability of lapsing within time t is highest for occasional, or erratic donors.

**Comparison of the survival estimates across groups:** under the log-rank test, the null hypothesis is that the survival estimates between groups are not different.

The trends exhibited by Kaplan-Meier curves are now analysed using the log-rank test and Peto-Peto test for comparing survival times between groups (Table 2).

**Table 2:** comparison of log-rank test and the Peto-Peto (ρ=1) test

Log-rank test			
**Characteristic**	**Chi-square**	**Degrees of freedom**	**p-value**
Gender	2.7	1	0.1
Age	13.9	1	0.0002
Interval between donations	5.6	1	0.02
Blood group	0	1	0.9
**Peto-Peto test results (ρ=1)**			
**Characteristic**	**Chi-square**	**Degrees of freedom**	**p-value**
Gender	2.6	1	0.1
Age	16.1	1	0.00006
Interval between donations	5.7	1	0.02
Blood group	0.1	1	0.8

The Peto-Peto test is now compared against the log-rank test to check the accuracy of the survival estimates. A parameter rho; ρ = 1 is used to select the Peto-Peto test.

The similarities in p-values between the log-rank test and the Peto-Peto test in Table 2 indicate that the blood donor survival (retention) estimates are valid. From Table 2, the results show that the survival (retention) estimates between male and female blood donors are not different (p-value = 0.1). From the results, there are significant differences between the survival (retention) curves for younger and older blood donors. The survival (retention) estimates between younger donors (< 40 years) and older donors (≥ 40 years) are different at a 5% level of significance (p = 0.0002). Results also show that the survival (retention) estimates between 3-month and 4-month intervals between donations are different (p-value = 0.02). The survival (retention) estimates between the universal blood group O and other blood groups are statistically not significant (p = 0.9), hence are not different.

**Univariate Cox PH:** Table 3 summarises the hazard ratio (HR) and their 95% Confidence Intervals (CI) for the survival (retention) curves above.

**Table 3:** hazard ratio (HR) for each donor characteristic from the univariate Cox proportional hazards (PH)

Characteristic	N	HR	95% CI	p-value
**Age**	450			
< 40 years		Basis		
≥ 40 years		0.5895	(0.4477; 0.7763)	0.000168
**Gender**	450			
Female		Basis		
Male		0.8031	(0.6209; 1.039)	0.095
**Blood group**	450			
Group O		Basis		
Other		0.9829	(0.7692; 1.256)	0.89
**Donation time interval**	450			
3 months		Basis		
4 months		1.386	(1.065; 1.804)	0.0152

Results in Table 3 show that the hazard of lapsing for blood donors (age ≥ 40 years) is 41% lower than for younger blood donors (age < 40 years). The 95% CI is (0.4477; 0.7763) with HR = 0.5895. The relationship between donor lapsing and age is statistically significant (p = 0.000168 < 0.05).

The hazard for lapsing in male donors is about 20% lower when compared to their female counterparts. The 95% CI for the HR is (0.6209; 1.039) with HR = 0.8031. The hazard for lapsing in other blood group donors is less than 2% lower when compared to their group O counterparts. The 95% CI for the HR is (0.7692; 1.256) with HR = 0.9829. The results also show that the hazard of lapsing for the 4-month intervals donors is 38.6% higher when compared to the donation time interval of 3 months. This means that, the longer the time a donor stays without donating blood, the higher the risk of lapsing. The hazard ratio of 1.386 is statistically significant with p-value = 0.0152 and 95% CI (1.065; 1.804) when not adjusting for other covariates.

The risk of lapsing for female blood donors is not statistically significant compared to male blood donors (p = 0.095). Similarly, the risk of lapsing from the other blood groups other than group O is not statistically significant (p-value = 0.89).

**Multiple Cox PH regression model:** based on expert judgment, clinical expertise, and statistical significance, a Cox PH model with age, interval between donations, and gender as covariates was considered. The likelihood ratio test was performed to compare models with covariates.

To test the effect of age, two Cox PH models were compared using the ANOVA function in R. H_0_: model 1: gender + time interval between donations; H_1_: model 2: age + gender + time interval between donations. Using the likelihood ratio test, the results are displayed in Table 4.

**Table 4:** analysis of deviance table

Model	Log likelihood ratio	Chi-square	df	p-value
1	1476.0			
2	1461.1	11.805	1	0.0005909

The two Cox PH models are statistically different (p-value= 0.0005909<0.05) and hence the larger model with age, gender, and time interval between donations was selected. The Cox PH model that explains the risk of lapsing among the blood donors in the donation pool can be presented as in Table 5.

**Table 5:** hazard ratios for donor characteristics based on multiple Cox proportional hazards (PH)

Characteristic	Hazard ratio (HR)	95% CI	p-value
**Age**			
< 40 years	Basis		
≥ 40 years	0.615	(0.461; 0.820)	0.000916
**Gender**			
Female Male	Basis		
	1.03	(0.537; 1.99)	0.922
**Donation time interval**			
3 months	Basis		
4 months	1.31	(0.667; 2.59)	0.430

Table 5 shows the results from the multiple Cox PH model which indicates that donor age has a significant effect on blood donor survival (retention) time (p=0.000918<0.05) with better survival (retention) in older donors (age ≥40 years). The older donors had a lower hazard (38.5% lower) compared to the hazard for younger donors. The effect of gender and donation time interval on donor survival was not statistically significant (p-values > 0.05). The C-statistic (concordance) of 0.6 shows that the model is more than just a random prediction.

The results in Table 6 show that all the covariates passed the individual proportionality test at the 0.05 level of significance (p-values > 0.05) and the model passed the global proportionality test at the 0.05 level (p-values = 0.65 > 0.05). Therefore, there is no statistical evidence to reject the null hypothesis and it is concluded that the risks are proportional over time.

**Table 6:** Cox proportional hazards (PH) assumption test

Covariate	Chi-square	Degrees of freedom	p-value
Age	1.56735	1	0.21
Gender	0.00143	1	0.97
Interval between donations	0.08507	1	0.77
Global	1.64551	3	0.65

## Discussion

This study complements the previous studies in expanding the body of knowledge through the application of survival analysis techniques in modelling time to blood donor lapsing in the case of a developing country, like Zimbabwe. Some studies have been conducted in the past to explain the effects of donor socio-demographic characteristics on blood donor lapsing and retention [11,23]. This study identified blood donor-specific factors or characteristics with an effect on donor lapsing during the four-year study period after the first-time donation. The findings from the study are beneficial to blood service authorities in decision making such as in blood donor mobilisation. The blood centres can develop better packages that are tailor-made for each niche of the donor category, all meant to attract and retain blood donors so that they make further donations. Blood centres often face the problem of donor attrition due to lapsing with many new donors lapsing within the first-year of their first-time donations [24,25]. This study confirmed the problem of donor attrition faced by blood centres with current study findings showing that 56.9% of the donors had lapsed by the end of the four-year study period. The study results are also consistent with findings from a related study in Brazil where it was established that a significant proportion of first-time donors rarely return for a second donation [11].

It is believed that as blood donors grow relatively older, they are more likely to donate blood more regularly compared to younger donors [26]. Other previous research results established that relatively older blood donors had lower lapsing risk compared to younger blood donors [27,28]. The findings from this study were consistent with these assertions with results showing that, the survival (retention) functions were not the same for younger and relatively older blood donors. The risk of lapsing between younger and older donors was statistically different. Younger donors had lower survival (retention) probabilities compared to the relatively older donors. At 20 months, as an example, the probability for survival (retention) was approximately 0.383 (38.3%) for younger donors and 0.571 (57.1%) for relatively older donors. This is partly due to the fact that an older blood donor has an altruistic motive and empathy towards blood donation. Relatively older donors take blood donation as a social responsibility that has to be religiously fulfilled when compared to young donors.

A study by Kheiri *et al*. [12] used an extended Cox proportional hazard model on the time interval between donations. The study established that, the donor's socio-demographic characteristics such as age, weight and occupation had significant effects on the time interval between donations. As an extension to their findings, the current study established that blood donors who took long intervals of time before making further blood donations, had a higher risk of lapsing. The current study also established that female young donors who had not yet reached menopause had a higher risk of lapsing compared to older female post-menopause and male donors.

Pertaining to the effect of gender on lapsing, the findings from the study concur with other researchers that there were no statistically significant differences in lapsing rates between male and female donors [16]. Even though the risk of lapsing between male and female blood donors was not statistically significant from the univariate analysis, other published studies showed that gender plays a critical role in donor motivation and return to further blood donation [29]. In contrast to the findings, other studies established significant differences for the gender covariate where female young donors had a higher risk of lapsing compared to their male counterparts [27,28,30]. These differences can be attributed to differences in geographical jurisdictions and cultural norms.

The simple Cox PH model shows that the risk of lapsing from other blood groups other than group O was not statistically significant. Clinical expertise assumed that blood group O was a universal blood group hence the demand for the blood group was usually higher thus prompting regular donations and lower lapsing risk. Contrary to this assumption, the findings in this study showed no significant differences between the blood group categories. The blood group factor was different from other previous studies where blood donors with other than the O blood group, had a high risk of lapsing [27,31].

## Conclusion

The application of the survival analysis techniques in this study is justifiable because of the existence of time-varying dependent variables, the presence of censoring, highly skewed data and the collected longitudinal data with each event recorded as it occurs over time. In other words, the application of survival analysis is relevant by considering the time between a fixed starting point (first-time blood donation) and a terminating event (donor lapsing). The skewed distribution of the survival data nullified the application of the normality assumption of linear regression. The presence of censoring again made linear regression an unsuitable way to analyse the data due biasness introduced by censoring, thus paving the way for the survival analysis techniques. In this article, one way to compare the association between blood donor characteristics and the hazard of donor lapsing was by examining the survival (retention) distributions using the Kaplan-Meier and Cox proportional hazard models. The use of expert judgement and clinical expertise played a crucial role in determining variables for the model. The study used the Kaplan-Meier plots to understand trends in the survival curves. The log-rank test was employed to quantify and compare the survival estimates between the subgroups of the donor covariates. To analyse the effect of the covariates on donor lapsing, Cox proportional hazards models were used. From the final model findings, the only variable that had a statistically significant effect on the hazard of lapsing was donor age. The blood supply in Zimbabwe is dependent upon mainly young first-time donors due to the high numbers of students recruited from high schools and institutions of higher learning. However, this gain in new donors is short-lived since many of the first-time donors lapse within the first twelve months of their donation career. Blood centre authorities need to develop radical intervention strategies that promote donor retention and encourage regular donations, especially from younger donors. The study findings contribute to the existing knowledge of blood donor retention. In Zimbabwe, the findings are that the problem of donor attrition exists, and donor age has a significant effect on blood donor retention, with relatively older donors less likely to lapse when compared to younger donors. Gender, blood donor group and donation time interval have no effect on donor retention or attrition. The analysis of blood donor lapsing undertaken here using survival analysis techniques extends the knowledge of blood donor management. The strengths of the current study include a randomised sample of subjects under study thereby, reducing chances of bias in conclusions. The availability of a complete record of data for all the variables used eliminated the need for data imputation. The main limitation of this study was access to all the blood donation data from the six blood centres under NBSZ. The study was therefore restricted to the headquarters blood centre as a representative of all the blood banks in the country. Basing the study at all the six blood centres could yield a more or better national outlook on blood donor retention and lapsing rates.

### What is known about this topic

- *Blood donor socio-demographic characteristics have an effect on blood donation behaviour;*
- *Blood donor retention is a challenge for many blood centres globally;*
- *Young blood donors constitute the greater proportion of the blood donor pool, yet they have higher attrition rates compared to older donors*.

### What this study adds

- *The study expanded the application of survival analysis techniques in modelling blood donor lapsing rates in a developing country like Zimbabwe;*
- *In the case of Zimbabwe, the findings are that the problem of donor attrition exists; the study findings confirm the challenge of blood donors lapsing, with 56.9% of the donors having lapsed by the end of the four-year study period; donor age has a significant effect on blood donor retention, with relatively older donors less likely to lapse when compared to their younger counterparts; gender, blood donor group and donation time interval have no statistically significant effect on donor retention or attrition;*
- *The knowledge of donor characteristics with influence on blood donation such as donor age and gender, helps in allocating resources during blood donor drives by targeting relevant groups of potential blood donors*.

## References

[1] Gillespie TW, Hillyer CD (2002). Blood donors and factors impacting the blood donation decision. Transfus Med Rev.

[2] Ownby HE, Kong F, Watanabe K, Tu Y, Nass CC (1999). Analysis of donor return behavior. Retrovirus Epidemiology Donor Study. Transfusion.

[3] Guo N, Wang J, Ness P, Yao F, Dong X, Bi X (2011). Analysis of Chinese donors' return behavior. Transfusion.

[4] Ou Y, Yau KK, Poon CM, Hui YV, Lee SS, Lee CK (2015). Donation frequency and its association with demographic characteristics-a 1-year observational study. Transfus Med.

[5] Singh AS, Dlamini S (2021). Analytical Models of Survival Analysis: Concepts and Their Applications. IIJSRM.

[6] Kachman SD (1999). Applications in survival analysis. J Anim Sci.

[7] Nemati M, Zhang H, Sloma M, Bekbolsynov D, Wang H, Stepkowski S (2021). Predicting Kidney Transplant Survival using Multiple Feature Representations for HLAs. Artif Intell Med Conf Artif Intell Med (2005-).

[8] Graham CN, Watson C, Barlev A, Stevenson M, Dharnidharka VR (2022). Mean lifetime survival estimates following solid organ transplantation in the US and UK. J Med Econ.

[9] Hamad F, Nezamoddin NK (2019). Potential Impact of Donors´ Factors on Survival Times of Transplanted Hearts and Lungs. Transplantation Reports.

[10] James R, Matthews D (1993). The donation cycle: a framework for the measurement and analysis of blood donor return behaviour. Vox Sang.

[11] Lourençon Ade F, Almeida RG, Ferreira O, Martinez EZ (2011). Evaluation of the return rate of volunteer blood donors. Rev Bras Hematol Hemoter.

[12] Kheiri S, Alibeigi Z (2015). An analysis of first-time blood donors return behaviour using regression models. Transfusion Med.

[13] Daneshi S, Davarani ER, Arefi F, Mehr FJ, Hushmandi K, Raei M (2021). Factors affecting blood donation intervals and patterns of return based on a sample in southern Iran: A follow-up design. Russian Open Medical Journal.

[14] James RC, Matthews DE (1996). Analysis of blood donor return behaviour using survival regression methods. Transfus Med.

[15] Melnyk SA, Pagell M, Jorae G, Sharpe A (1995). Applying survival analysis to operations management: Analyzing the differences in donor classes in the blood donation process. Journal of Operations Management.

[16] Gemelli CN, Hayman J, Waller D (2017). Frequent whole blood donors: understanding this population and predictors of lapse. Transfusion.

[17] Israel GD (1992). Determining Sample Size.

[18] Clark TG, Bradburn MJ, Love SB, Altman DG (2003). Survival analysis part I: basic concepts and first analyses. Br J Cancer.

[19] Yen-Chi C (2018). Introduction to Nonparametric Statistics Instructor: Lecture 5: Survival Analysis.

[20] Etikan I, Abubakar S, Alkassim R (2017). The kaplan meier estimate in survival analysis. Biom Biostat Int J.

[21] Bradburn MJ, Clark TG, Love SB, Altman DG (2003). Survival analysis part II: multivariate data analysis--an introduction to concepts and methods. Br J Cancer.

[22] Therneau TM (2000). Modelling survival data: extending the Cox model. Springer.

[23] Burgdorf KS, Simonsen J, Sundby A, Rostgaard K, Pedersen OB, Sørensen E (2017). Socio-demographic characteristics of Danish blood donors. PLoS One.

[24] Schreiber GB, Sharma UK, Wright DJ, Glynn SA, Ownby HE, Tu Y (2005). First year donation patterns predict long-term commitment for first-time donors. Vox Sang.

[25] Almeida-Neto C (2011). Retention of blood donors: Strategies to fulfill the requirements of blood centers. Rev Bras Hematol Hemoter.

[26] Davison TE, Masser BM, Thorpe R (2019). Growing evidence supports healthy older people continuing to donate blood into later life. Transfusion.

[27] Royse D, Doochin KE (1995). Multi-gallon blood donors: who are they?. Transfusion.

[28] Germain M, Glynn SA, Schreiber GB, Gélinas S, King M, Jones M (2007). Determinants of return behavior: a comparison of current and lapsed donors. Transfusion.

[29] Bani M, Giussani B (2010). Gender differences in giving blood: a review of the literature. Blood Transfus.

[30] Weidmann C, Müller-Steinhardt M, Schneider S, Weck E, Klüter H (2012). Characteristics of Lapsed German Whole Blood Donors and Barriers to Return Four Years after the Initial Donation. Transfus Med Hemother.

[31] Veldhuizen IJT, Doggen CJM, Atsma F, De Kort WLAM (2009). Donor profiles: demographic factors and their influence on the donor career. Vox Sang.

